# A Simple Intervention to Improve Equity in Obstetric Research

**DOI:** 10.1089/heq.2023.0073

**Published:** 2023-10-16

**Authors:** Rebecca Feldman Hamm, Meaghan G. McCabe, Abike James, Samuel Parry, Lisa D. Levine

**Affiliations:** ^1^ Department of Obstetrics and Gynecology, University of Pennsylvania Perelman School of Medicine, Philadelphia, Pennsylvania, USA.; ^2^ Leonard Davis Institute of Health Economics, Perelman School of Medicine, Philadelphia, Pennsylvania, USA.

**Keywords:** equity, research, obstetrics, approach, recruitment

## Abstract

**Introduction:**

To evaluate if a simple intervention, including formation of a Research Equity Committee and a dashboard detailing study approach and enrollment statistics by race, could improve equitable inclusion in obstetric research.

**Methods:**

Our intervention had four components: (1) research personnel submitted dashboards every 3 months to the Research Equity Committee; (2) approach and enrollment by race were compared with expected racial breakdown; (3) study teams with rates of approach and/or enrollment of black birthing people below goal met with the committee for root cause analysis (RCA) and action planning; (4) all dashboards, RCAs, and action plans were presented at 3-month intervals. We prospectively evaluated the impact of this intervention on the inclusion of self-reported black birthing people in actively enrolling obstetrical studies at an academic university from July 2021 to June 2022.

**Results:**

Seven qualifying prospective studies submitted 23 equity dashboards, which encompassed 692 patients. Six RCAs and action planning were held. Themes of developed action plans included: (1) standardizing how, when, and which patients to approach to eliminate approach bias, (2) standardized scripts for patient recruitment, and (3) study expansion to more diverse clinics. All four studies that underwent an RCA demonstrated improvements after the intervention; however, only one study demonstrated a statistically significant increase in approach (*p*=0.002) and enrollment (*p*=0.02) of black birthing people across the study period.

**Discussion and Health Equity Implications:**

A simple intervention can improve approach and enrollment of black birthing people in obstetric research.

## Introduction

Profound racial and ethnic disparities are present in most obstetric outcomes, including maternal and neonatal morbidity and mortality.^1,2^ Obstetric research is performed to generate evidence to improve these outcomes. Yet, the marginalized populations most at need of evidence-based practices in maternity care are often those least represented in clinical studies, with deficient representation of American Indian or Alaskan Native, Asian, black, and Latinx populations.^3,4^

To meet the needs of all populations at risk of poor health outcomes, it is imperative to increase diversity and inclusion in health care research endeavors.^5^ At the request of Congress, the National Academies of Sciences, Engineering, and Medicine produced an expansive report detailing 17 recommendations to improve representation in clinical research, such as increased accountability.^6^ Specific to women's health, in the Committee Opinion on Ethical Considerations for Including Women as Research Participants, the American College of Obstetricians and Gynecologists (ACOG) states that “efforts are needed to ensure that research is designed to include representation of all potentially affected individuals, including those in diverse and underserved populations who often are not fully represented in current study designs.”^7^

Yet, there is a paucity of data for investigators and research teams in how best to accomplish the important goal of performing inclusive research. A recent Society for Maternal Fetal Medicine statement suggests an antiracist framework for approaching race in obstetric research.^8^

In response, our group took a race-conscious approach to improving research inclusivity in obstetric studies, forming a Research Equity Committee and developing a dashboard detailing study approach and enrollment statistics by race and ethnicity. In this study, we aimed to describe this intervention and determine if this work could improve equitable research inclusion across an academic obstetric research program.

## Methods

We performed a prospective study evaluating the impact of a race-conscious intervention on the inclusion of self-reported black birthing people in actively enrolling obstetrical studies in the context of a centralized research program at a single academic university from July 2021 to June 2022. The project was determined to qualify as quality improvement by the University of Pennsylvania Perelman School of Medicine Institutional Review Board. STROBE guidelines and the Green Journal equity rubric were utilized in article preparation.^9^ In conceptualizing our process and findings, we considered its relationship to the 5Ws of Racial Equity in Research, a framework for applying a racial equity lens throughout the research process.^10^ The 5Ws of Racial Equity in Research framework use Who, What, When, Where, and Why to offer simple and relatable guidance (the how) for promoting racial equity in research processes and among the research workforce.

The centralized research program encompasses diverse types of research, from basic to translational to clinical research and is supported by both intramural and extramural funding. This research program benefits from collaborations across diverse disciplines (e.g., neonatology, immunology, microbiology, molecular genetics, cardiology, perinatal pathology, molecular biology, pharmacology, metabolic disorders) to advance scientific pursuit, and often has more than 20 ongoing clinical studies at a time. There is a concerted effort to diversify the research workforce at our institution, with the perspective that race concordance among research staff and the patient population will benefit design, processes, enrollment, and retention. At that time of this article, 50% of our research staff (*n*=9/18) identified as Black, Indigenous, or People of Color.

Within this research program, a Research Equity Committee was established in May 2021. The committee included a senior maternal fetal medicine (MFM) physician scientist with experience in basic and translational research, an MFM physician scientist focused on equity, and a lead program manager of the research program.

**FIG. 1.**
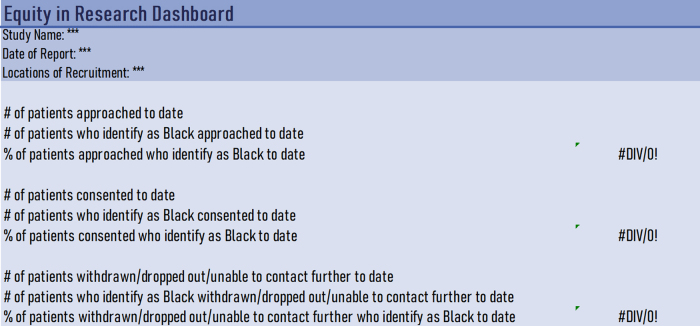
Example of “Equity in Research” dashboard. Example shown specifically for black race; similar dashboards were submitted by research staff for Latinx ethnicity and public insurance.

The developed intervention had four components. First, research personnel for each actively enrolling study within the centralized research program were asked to submit an “Equity in Research” dashboard at 3-month intervals to the Research Equity Committee. This dashboard detailed study approach, enrollment, and retention overall and since the last dashboard submission for patients of black race, Latinx ethnicity, and public insurance. Race and ethnicity were pulled from self-report identified in the electronic health record. An example of this dashboard is shown for patients of black race in Figure 1.

Second, after each dashboard submission date (labeled as Checkpoints #1–4), dashboards were reviewed by the Research Equity Committee. Approach and enrollment by race were compared with an expected representation of black birthing people, with an overall goal of 60% black birthing patients based on the racial breakdown in site demographics. We focused the committee meetings on inclusion of black birthing people due to a high prevalence in our site population (∼60%) and the severity of disparities in maternal morbidity and mortality in this specific population.^11^ Studies not meeting the approach goal of >60% black birthing people were selected for root cause analysis (RCA). Studies that had a previous RCA could be eligible for a second if representation goals remained unreached.

As the third component of the intervention, the committee scheduled RCA meetings with the selected study teams (those not meeting the approach goal of >60% black birthing people). Meetings included primary investigators, project managers, and clinical research coordinators and were held in the month following dashboard submission. In these meetings, the committee members reviewed the submitted dashboard with the team, discussing approach and enrollment of black birthing people in the context of that specific study, to uncover possible barriers and facilitators to equitable inclusion.

The 5Ws of Racial Equity in Research framework were reflected in these conversations.^10^ For example, discussion questions included “what resources and accommodations are needed such that engagement in the study comes at minimal burden and maximum benefit to participants” and “where is the research occurring, and how is this impacting equitable inclusion?” Research teams, along with the committee, developed concrete action plans during these meetings to improve study inclusivity.

**FIG. 2.**
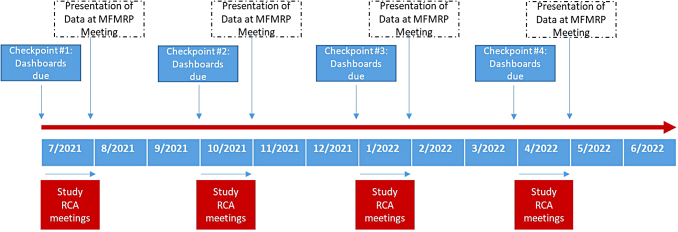
Time line of study events throughout study period, July 2021 to June 2022.

As the fourth component of the intervention, all dashboards, RCAs, and action plans were presented by the Research Equity Committee to all research faculty and staff 1 month after each dashboard submission date during a regularly scheduled monthly program-wide research meeting. Figure 2 details this time line of events over the 1-year study period.

As there were obstetric studies that began or ended during the study period, studies that submitted more than one “Equity in Research” dashboard to the Research Equity Committee were included for analysis. Cochran–Armitage tests were used to evaluate for trends in approach and enrollment of black birthing people for each included study over time. Statistical analyses were performed with Stata 15 (StataCorp, College Station, TX, USA). All tests were two-tailed, and *p*-values <0.05 were considered statistically significant.

## Results

There were 11 ongoing prospectively enrolling studies in the centralized research program during the study period. Of these, four submitted only one “Equity in Research” dashboard to the Research Equity Committee, and were excluded from analysis. Of the four excluded studies, two randomized clinical trials (RCTs) concluded in the initial 3 months of the study period, one was a small cohort study that crossed only one dashboard submission date before completion, and one cohort study began in the final 3 months of the study period. Seven studies crossed more than dashboard submission Checkpoint to the committee and were included for analysis. Of these, three were RCTs and four cohort studies; the studies varied in size (range *n*=27–314) and clinical topic within obstetrics, including studies on pain management after cesarean delivery, COVID-19 in pregnancy, and treatments for short cervical length.

Among those 7 studies, 23 dashboards were submitted during the study period, encompassing information on 692 patients approached for research. Further information regarding these studies can be found in Table 1.

**Table 1.** Characteristics of Included Studies

Study no.	Type	Clinical topic	Inpatient/outpatient recruitment	Anticipated enrollment^a^	No. of equity dashboards submitted during study period	RCA held (Y/*N*)
1	RCT	Pain management	Inpatient	300	4	Y
2	Cohort	COVID-19	Inpatient	200	4	Y
3	RCT	Preterm birth	Outpatient	100	4	Y
4	RCT	Preterm birth	Outpatient	100	3	N
5	RCT	Preeclampsia	Outpatient	180	2	N
6	Cohort	Twin pregnancies	Outpatient	50	3	Y
7	Cohort	Fetal growth restriction	Inpatient and outpatient	100	2	N

^a^ If multisite study, anticipated enrollment is for University of Pennsylvania only.

RCA, root cause analysis; RCT, randomized controlled trial.

During the study period, four of the seven studies qualified for RCA based on our predetermined criteria and six RCAs were held (two of these studies qualified for a repeat RCA at a second Checkpoint). During these RCAs, possible contributors to lower than expected rates of approach and enrollment of black birthing people were identified and concrete action plans developed. For example, Study #2 experienced a drop in the approach of black birthing people at Checkpoint #3. The RCA found that staffing limitations were preventing all eligible participants from being approached, leaving room for approach bias. Action planning developed standardized processes for approach; by Checkpoint #4, diversity in approach improved.

Other action plans that aimed to improve inclusion of black birthing people included standardized scripting for patient recruitment, considering how social determinants of health might make it difficult for patients to participate in a given study protocol, and study expansion to more racially diverse sites.

**FIG. 3.**
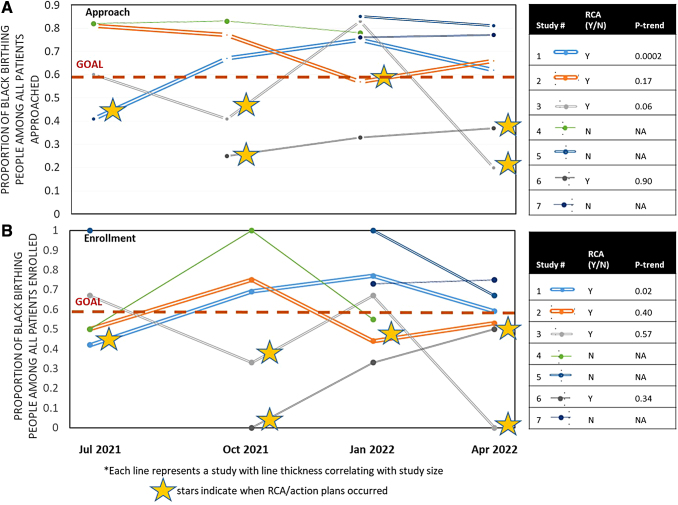
**(A)** Approach and **(B)** enrollment of black birthing people in obstetric research at one academic institution, by study, after implementation of a race-conscious intervention from July 2021 to June 2022. Legends indicate whether or not RCA occurred, and *p*-trend for inclusion of black birthing people over time across the study period. RCA, root cause analysis.

Figure 3 demonstrates rates of approach (3A) and enrollment (3B) of black birthing people by study after the initiation of our research program's equity-focused intervention. All four studies that underwent an RCA demonstrated improvements after the intervention; however, only one study demonstrated a statistically significant increase in approach (*p*=0.002) and enrollment (*p*=0.02) of black birthing people across the study period. The three studies that did not undergo RCA remained above goal for approach and enrollment of black birthing people throughout the study period. We were unable to evaluate for trends in study retention due to the overall low dropout rates across studies.

## Discussion

In this study, our research program developed a race-conscious approach to performing obstetric research, including formation of a Research Equity Committee and a dashboard detailing study approach and enrollment statistics by race. Utilization of this intervention demonstrated improved approach and enrollment of black birthing people during the 1-year study period.

The call for action to improve inclusion of marginalized population in health care research is strong and resounding.^6–8^ Yet, the path to reaching this goal remains unclear. For example, the National Institute of Health (NIH) has implemented initiatives designed to foster the inclusion of underrepresented groups in NIH-supported clinical research trials, including structured reporting systems; despite this initiative, underrepresentation of diverse groups in clinical trials persists.^12^ In other mixed-methods work designed to elucidate facilitators to recruitment and retention strategies of underrepresented groups, several themes emerged, including: (1) starting with an intention to achieve representativeness, (2) establishing a foundation of trust with participants and community, and (3) anticipating and removing barriers to study participation.^6^ Yet, many of these studies offering recommendations do not address the effectiveness of each suggested intervention.

Our work combines a reporting system for racial and ethnic inclusion in ongoing studies, similar to what has been done through the NIH, with an individualized approach to overcoming barriers and selecting needed facilitators targeted to improving equitable inclusion in a given research study. Furthermore, this work begins to provide an evidence base for an intervention that may move the needle on research representation.

Our study is not without limitations. First, this work was performed in the context of a large research program at one academic institution, and is thus limited in its generalizability. Several of the studies included in this work were either of a small sample size overall, or recruited slowly during the study period. In these studies, we were likely underpowered to evaluate for statistically significant changes in approach and enrollment. Our study also focused specifically on inclusion of black birthing people, rather than other marginalized groups. This initial goal was intentionally selected at our institution given the large black population we serve, the known disparities in maternal and neonatal outcomes, and a recognition that this was a specific area in which we needed to improve.

However, future program goals will be to improve the inclusion of all Black, Indigenous, and People of Color in obstetric research. Notably, effective interventions for improving equitable inclusion may change based on the population being considered, such as considering whether recruitment or consent materials are available in non-English languages. Finally, the small number of patients who withdrew from studies during our study period made it difficult to evaluate equity in study retention.

## Health Equity Implications

Interventions such as ours could be refined and replicated for individual studies, other institutional research programs, or even within national research consortiums. Such interventions would be ripe for further research. What components of the intervention are most effective? Are there evidence-based cross-cutting themes among action plans that improve equitable approach and/or enrollment? Could such plans be initiated at study starts rather than awaiting issues? Can patient representation and/or the community voice on the Research Equity Committee add even further value to its effect?

There is an imperative for increasing diversity in health care research endeavors, especially given the profound racial and ethnic disparities in maternal and neonatal morbidity. This simple intervention may begin to improve the approach and enrollment of populations most at need of effective interventions in obstetric research.

## Abbreviations Used

MFM: maternal fetal medicine
NIH: National Institute of Health
RCA: root cause analysis
RCT: randomized clinical trial
